# An evaluation of gout visits in the United States for the years 2007 to 2011

**DOI:** 10.1186/s41927-018-0020-0

**Published:** 2018-05-30

**Authors:** Kristen E. Castro, Kaitlyn D. Corey, Diana L. Raymond, Michael R. Jiroutek, Melissa A. Holland

**Affiliations:** Campbell University College of Pharmacy & Health Sciences, 180 Main Street PO Box 1090, Buies Creek, NC 27506 USA

**Keywords:** Gout, Febuxostat, NAMCS, NHAMCS-OPD, NHAMCS-ED

## Abstract

**Background:**

This study analyzed visits for and factors associated with gout and gout medication treatment trends for the years 2007–2011 in the United States given the introduction of febuxostat, the first new treatment option for gout in over 40 years, which was introduced to the market in 2009.

**Methods:**

This study was a retrospective, cross-sectional, observational study of patients age 20 and older seen by providers who participated in the National Ambulatory Medical Care Survey (NAMCS), the National Hospital Ambulatory Medical Care Survey Outpatient Department (NHAMCS-OPD) or Emergency Department (NHAMCS-ED) in the United States. The outcome of interest was visits for gout diagnosis and visits where a gout medication was prescribed.

**Results:**

Approximately 1.2% of visits had a diagnosis of gout. There was a significant increase in the percentage of visits with a diagnosis of gout in years 2009–2011 compared to 2007–2008, which remained after adjusting for covariates of interest. Groups more likely to have a visit with gout included those ≥65 and 45–64 (both as compared to those 20–44), the African-American and ‘Other’ race groups (as compared to Caucasians) and those on a diuretic. Groups less likely to have a visit with gout included females, Hispanic/Latinos, those with insurance type of ‘Other’ and Medicaid (both as compared to private insurance) and visits to a hospital emergency setting (as compared to physician’s office visits).

**Conclusion:**

Although there was a significant increase in visits where gout is diagnosed across study years, the overall percentage of visits with a gout diagnosis is low in the US population. Treatment trends over the study years has remained consistent, with the introduction of febuxostat appearing to have little impact for the study years through 2011.

## Background

Gout is a type of inflammatory arthritis associated with the formation of urate crystals in the joints. It is estimated that approximately 4% of Americans are affected, but previous research has shown the prevalence of gout is increasing [1]. Contributing factors to gout include increase in obesity, hypertension, and purine-rich diets [2]. The severity and progression of gout has been directly correlated to an increase in age. [3] Gout is known to be more predominant in males, but is seen in postmenopausal women. The prevalence of gout is higher in African Americans as compared to Caucasians, with increasing prevalence overall across all demographics [3]. Prior to 2009, pharmacological treatment options for gout had not changed in many years. A new treatment option, febuxostat, was approved by the Food and Drug Administration in February 2009 [4].

No studies have evaluated the proportion of visits with a gout diagnosis since 2009 when febuxostat was introduced to the market. Previous studies have shown that an above normal BMI, hypertension, dyslipidemia, use of a diuretic and older age are associated with an increased risk of gout [5–10].

With the first new gout therapy in over 40 years introduced to the market in 2009, this study sought to evaluate changes in gout-related ambulatory and emergency department visits for the years 2007 through 2011 (the most recent data available) as well as assess factors associated with gout [11]. Additionally, gout treatment trends were plotted to determine the impact of febuxostat on gout therapy since its introduction to the market.

## Methods

This retrospective, cross-sectional, observational study analyzed data collected in the National Ambulatory Medical Care Survey (NAMCS) and the National Hospital Ambulatory Medical Care Survey Outpatient Department & Emergency Department (NHAMCS-OPD/NHAMCS-ED) during the years 2007–2011. Hundreds of reports, manuscripts and books based on data from these widely utilized and respected surveys have been published since the 1970s (https://www.cdc.gov/nchs/data/ahcd/namcs_nhamcs_publication_list.pdf).

The NHAMCS is an annual, national probability sample of ambulatory visits made to non-federal, general, and short-stay hospitals in the US conducted by the Centers for Disease Control and Prevention, National Center for Health Statistics (NCHS). The multi-staged sampling design is composed of four stages and includes visits to both selected emergency care departments as well as hospital outpatient departments [12–14]. The NAMCS is an annual, national probability sample of visits made to the offices of non-federally employed physicians classified by the American Medical Association or the American Osteopathic Association as “office-based, patient care” (excluding anesthesiologists, pathologists and radiologists) [12–14]. For more information regarding the survey instruments, scope and sample design, data collection and processing, estimation procedures and reliability of survey estimates, go to http://www.cdc.gov/nchs/ahcd/ahcd_questionnaires.htm.

NAMCS, NHAMCS-OPD and NHAMCS-ED datasets covering five years (2007–2011) were included in this study. Patients 20 or older from any of the three databases were coded as included in the final analysis dataset. There were no exclusions for the study. Across all five years a total of 128,734 raw records in the NHAMCS-OPD, 126,836 raw records in the NHAMCS-ED and 126,651 in the NAMCS databases met the inclusion criteria (382,221 combined). The study was submitted to the Campbell University Institutional Review Board and received an exemption due to the data sources used being publicly available and de-identified. As such, since this research was based solely on the analysis of previously collected, de-identified data, it complies with the Helsinki Declaration.

The survey data were analyzed using the sampled visit weight that is the product of the corresponding sampling fractions at each stage in the sample design. The sampling weights have been adjusted by NCHS for survey nonresponse as appropriate within each database, yielding unbiased national annual estimates of visit occurrences, percentages, and characteristics [12].

Because of the complex sample design, sampling errors were determined using the SAS SURVEYFREQ and SURVEYLOGISTIC procedures which take into account the clustered nature of the sample [15]. The appropriate NOMCAR and DOMAIN statements/options were implemented in these procedures as recommended by the NCHS [12].

The dependent variable of interest was a diagnosis of gout, where the denominator is the number of visits meeting the inclusion/exclusion criteria. A diagnosis of gout was defined by the appropriate diagnosis codes found in any of the DIAG1-DIAG3 diagnosis fields or appropriate gout medication codes for allopurinol, febuxostat, colchicine, probenecid, and colchicine-probenecid found in any of the DRUGID1-DRUGID8 medication fields.

Rao-Scott chi-square tests were used to analyze whether the proportion of visits with a diagnosis of gout differs by year group (2007–2008 vs. 2009–2011) and whether any association exists between visits with a diagnosis of gout and each of the following variables: age, sex, race, ethnicity, region (US geographic regions included Northeast, Midwest, South, and West), metropolitan statistical area (MSA), insurance status (private, Medicaid or Children’s Health Insurance Program [CHIP]/State Children’s Health Insurance Program [SCHIP], Medicare, and other [worker’s compensation, self-pay, no charge/charity, other], setting type (physician office, hospital outpatient department, hospital emergency department) and diuretic use. These variables were grouped for analysis as shown in Table 1. Odds ratios (ORs), corresponding 95% confidence intervals (CIs) and *p*-values were reported.

**Table 1:** Demographics and Patient Characteristics (*N* = 382,221)^a^

Characteristic	No. (%) of Patient Visits^b^
Observation Period	
2009–2011	588,791,594 (61)
2007–2008	375,839,802 (39)
Age (years)	
Mean (SE)	53.9 (0.23)
Age Group	
≥65	299,032,423 (31)
45–64	351,209,142 (36)
20–44	314,389,831 (33)
Sex	
Female	587,394,025 (61)
Male	377,237,371 (39)
Race	
Other	33,516,436 (5)
African-American	99,604,070 (14)
Caucasian	602,374,076 (82)
Ethnicity	
Hispanic/Latino	79,262,491 (11)
Non-Hispanic/Latino	630,345,157 (89)
Region	
Northeast	189,690,837 (20)
Midwest	206,775,296 (21)
West	201,670,759 (21)
South	366,494,504 (38)
MSA	
Non-MSA	120,846,147 (13)
MSA	843,785,249 (87)
Insurance Status	
Other	98,736,670 (11)
Medicaid	88,374,398 (10)
Medicare	280,902,288 (30)
Private Insurance	461,730,141 (49)
Setting Type	
Hospital Emergency	94,712,528 (10)
Hospital Outpatient	78,137,131 (8)
Physician’s Office	791,781,738 (82)
Diuretic Use	
Yes	71,243,249 (7)
No	893,388,147 (93)
Gout	
Yes	11,769,697 (1)
No	952,861,700 (99)

*MSA* Metropolitan Statistical Area

^a^Unweighted, raw study sample size

^b^Survey weighting and clusters accounted for reflecting unbiased, national annual estimates of visit occurrences for the portion of the population meeting the study inclusion/exclusion criteria

A multivariable logistic regression model was also constructed in order to evaluate the predictive value of all the independent variables of interest simultaneously on visits with a diagnosis of gout, adjusting for covariates of interest. As a primary model filter, only variables with an overall chi-square test of association *p*-value < 0.2 were included in the multivariable model (year group was included regardless). ORs with corresponding 95% CIs and *p*-values for each level of each variable included in the model (in comparison to each variable’s reference group) were reported. No collinearity issues between the independent variables included in the model were found. All analyses were generated using SAS software, version 9.3. Plots of the percentage of visits per year with gout medication by drug class and individual drug were constructed to descriptively assess gout treatment trends.

Per NCHS recommendations, any variable with a survey estimate based on either less than 30 records, a relative standard error of more than 30%, or more than 30% missing data was excluded from the analyses due to potential unreliability [12]. As a result, the variables of interest weight status, tobacco use, depression, hypertension, and diabetes were excluded from all analyses due to not meeting one or more of the above listed criteria. Missing values were treated as missing in the statistical evaluation. No adjustments for multiple comparisons were made and *p*-values < 0.05 were considered statistically significant.

## Results

During the study period (2007–2011), the NAMCS, NHAMCS-OPD, and NHAMCS-ED datasets include a total of 495,370 patient visits (unweighted, raw data). A total of 382,221 patient visits within this five-year period met the inclusion criteria and were included in this study (Table 1). Most variables had no missing data, however, ethnicity, race and insurance status were missing 21, 16 and 5%, respectively. Gout was diagnosed in just over 1.2% of all patient visits. More patient visits occurred during the more recent half of the study period (61% in the years 2009–2010). The mean age (SE) was 53.9 (0.23) years, with a similar percentage of patients in each of the three age groups. Of the patient visits included in the analyses, 61% were female, 82% were Caucasian, 14% were African-American, and 11% were Hispanic/Latino. Nearly twice as many visits occurred in the South (38%) as compared to the other regions (21% in the Midwest, 21% in the West and 20% in the Northeast). However, visits in metropolitan areas represented 87% of the study total. Private insurance was presented at the majority of all patient visits (50%), with Medicare presented at 30% of visits and Medicaid at 10%. The vast majority of visits occurred in a physician’s office (82%), reflecting the NAMCS survey data (collected in physician offices). Only 7% of patient visits reported diuretic use.

The primary analysis showed a 30% relative increase in the percentage of patients who had a visit with a diagnosis of gout in the years 2009–2011 compared to 2007–2008 (1.3% vs. 1.0%, respectively; OR 1.28, 95% CI 1.10–1.49) (Table 2). The individual chi-square tests of the other covariates of interest that comprised the first part of the secondary analysis, showed a significant association between visits with a diagnosis of gout and the following variables: age, sex, ethnicity, race group, insurance status, setting type, region and diuretic use. See the univariable columns in Table 2 for the details of these individual associations with visits for a diagnosis of gout.

**Table 2:** Gout Predictor Variables, Univariable and Multivariable Analyses* Data are given as number (%) of patients

			Univariable		Multivariable	
Parameter	Gout (%)	No Gout (%)	OR (95% CI)	P	OR (95% CI)	P
Observation Period						
2009–2011	7,849,421 (1.3)	580,942,174 (98.7)	1.28 (1.10–1.49)	0.0010	1.24 (1.02–1.52)	0.0346
2007–2008	3,920,276 (1.0)	371,919,526 (99.0)	Referent	--	Referent	--
Age Group (years)						
≥ 65	7,113,034 (2.4)	291,919,369 (97.6)	8.64 (7.04–10.60)	< 0.0001	4.94 (3.69–6.62)	< 0.0001
45–64	3,772,290 (1.1)	347,436,851 (98.9)	3.85 (3.11–4.77)	< 0.0001	2.52 (1.97–3.21)	< 0.0001
20–44	884,372 (0.3)	313,505,459 (99.7)	Referent	–	Referent	–
Sex						
Female	3,055,558 (0.5)	584,338,467 (99.5)	0.22 (0.20–0.25)	< 0.0001	0.20 (0.18–0.24)	< 0.0001
Male	8,714,139 (2.3)	368,523,233 (97.7)	Referent	–	Referent	–
Race						
Other	574,306 (1.7)	32,942,130 (98.3)	1.43 (1.03–1.99)	0.0332	2.02 (1.47–2.77)	< 0.0001
African-American	1,226,437 (1.2)	98,377,632 (98.8)	1.02 (0.82–1.27)	0.8513	1.33 (1.03–1.72)	0.0271
Caucasian	7,264,368 (1.2)	595,109,707 (98.8)	Referent	--	Referent	--
Ethnicity						
Hispanic/Latino	511,614 (0.6)	78,750,878 (99.4)	0.49 (0.36–0.67)	< 0.0001	0.64 (0.48–0.86)	0.0032
Non-Hispanic/Latino	8,252,946 (1.3)	622,092,211 (98.7)	Referent	–	Referent	–
Region						
Northeast	2,019,734 (1.1)	187,761,102 (98.9)	0.90 (0.72–1.13)	0.3629	0.80 (0.62–1.03)	0.0823
Midwest	3,053,963 (1.5)	203,721,333 (98.5)	1.25 (1.04–1.52)	0.0169	1.16 (0.95–1.43)	0.1501
West	2,367,739 (1.2)	199,303,020 (98.8)	0.99 (0.80–1.24)	0.9580	0.86 (0.67–1.10)	0.2296
South	4,328,261 (1.2)	362,166,243 (98.8)	Referent	--	Referent	--
MSA						
Non-MSA	1,709,090 (1.4)	119,137,057 (98.6)	1.18 (0.93–1.52)	0.1636	1.01 (0.77–1.32)	0.9462
MSA	10,060,607 (1.2)	833,724,642 (98.8)	Referent	--	Referent	--
Insurance Status						
Other	407,967 (0.4)	98,328,703 (99.6)	0.43 (0.32–0.58)	< 0.0001	0.51 (0.36–0.72)	0.0001
Medicaid	407,549 (0.5)	87,966,848 (99.5)	0.48 (0.35–0.66)	< 0.0001	0.56 (0.39–0.80)	0.0014
Medicare	6,193,910 (2.2)	274,708,378 (97.8)	2.35 (2.07–2.68)	< 0.0001	0.93 (0.74–1.15)	0.4846
Private Insurance	4,382,477 (0.9)	457,347,665 (99.1)	Referent	–	Referent	–
Setting Type						
Hospital Emergency	353,199 (0.4)	94,359,329 (99.6)	0.28 (0.23–0.33)	< 0.0001	0.42 (0.34–0.51)	< 0.0001
Hospital Outpatient	892,347 (1.1)	77,244,784 (98.9)	0.86 (0.71–1.03)	0.0994	1.05 (0.88–1.26)	0.5718
Physician Office	10,524,151 (1.3)	781,257,587 (98.7)	Referent	–	Referent	–
Diuretic Use						
Yes	3,575,442 (5.0)	67,667,807 (95.0)	5.71 (5.04–6.47)	< 0.0001	4.00 (3.37–4.75)	< 0.0001
No	8,194,254 (0.9)	885,193,893 (99.1)	Referent	–	Referent	–

*MSA* Metropolitan Statistical Area

* Survey weighting and clusters accounted for reflecting unbiased, national annual estimates of visit occurrences for the portion of the population meeting the study inclusion/exclusion criteria

Note that per the model fitting criterion described in the methods section, no variables were excluded from the multivariable model

The weighted multivariable logistic regression model, allowing adjustment for the effect of potentially important variables, demonstrated that significant associations remained between visits with a gout diagnosis and age group (≥65 and 45–64 vs. 20–44), sex, ethnicity, race group (‘Other’ vs. Caucasian as well as now African-American vs. Caucasian), insurance status (Other and Medicaid vs. private insurance, but no longer Medicare vs. private insurance), hospital emergency departments (vs. physician office visits) and diuretic use. However, the associations between visits with a gout diagnosis and region^1^ of the country and as well as gout visits and Medicare (vs. private insurance) did not remain significant following adjustment for the effect of the other covariates of interest in the model. A diagnosis of gout remained more likely for patient visits in 2009–2011 as compared to visits in 2007–2008 (OR 1.24, 95% CI 1.02–1.52). Additionally, a diagnosis of gout remained more likely for visits with patients 65 and older and patients 45–64 as compare to visits for those 20–44 (OR 4.94, 95% CI 3.69–6.62 and OR 2.52, 95% CI 1.97–3.21, respectively). Gout was less likely to be diagnosed at visits for females as compared to males (OR 0.20, 95% CI 0.18–0.24) and Hispanic/Latino patients as compared to Non-Hispanic/Latino patients (OR 0.64, 95% CI 0.48–0.86), while gout was more likely to be diagnosed at visits for those on a diuretic as compared to those not on a diuretic (OR 4.00, 95% CI 3.37–4.75). Gout was less likely to be diagnosed at patient visits with an insurance status of “Other” and Medicaid as compared to those with private insurance (OR 0.51, 95% CI 0.36–0.72 and OR 0.56, 95% CI 0.39–0.80, respectively). Patients whose visit was recorded in an emergency department were less likely (OR 0.42, 95% CI 0.34–0.51) to be diagnosed with gout as compared to those with a physician’s office visit (Table 2). Second order interaction terms were investigated, found to contribute nothing significant to the understanding of the overall results and were excluded from the final reported model.

**Fig. 1:**
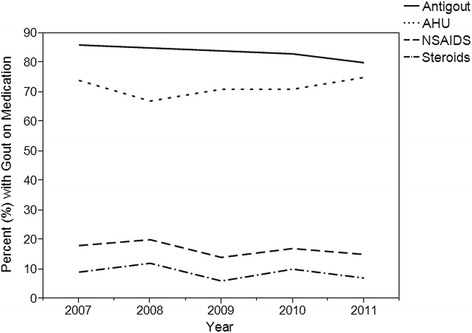
Gout Prescription Trends by Drug Class, 2007–2011

**Fig. 2:**
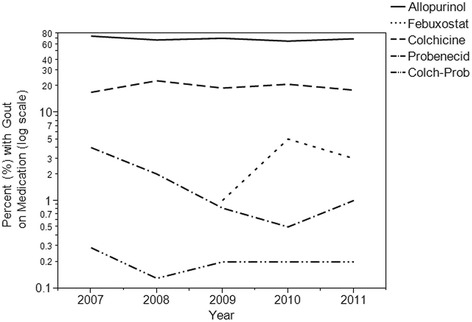
Gout Prescription Trends by Individual Drug, 2007–2011

Finally, plots of the percentage of patient visits with gout by year for medication class and individual drug showing the most common gout treatments can be seen in Figs. 1 and 2. The percent of gout visits in each drug class appears similar across the years studied. Antigout (80–86%) and antihyperuricemic (67–75%) medications were consistently the most common treatment while NSAIDs (14–20%) and steroids (6–12%) were a distant third and fourth, respectively.

Among individual medications, allopurinol (67–74%) is clearly the most common, three to four times as likely as the next closest (colchicine). Febuxostat, probenecid and colchicine-probenecid were infrequently prescribed (all less than 5%). Allopurinol and colchicine dominate the market, their use appears consistent across the years studied, while probenecid’s use has decreased over time. Uptake does appear to be slow for febuxostat, the newest gout medication, as two years after entering the market, the percentage of patients with gout taking the drug remains around 3%.

## Discussion

The proportion of patient visits with a diagnosis of gout increased between 2007 and 2011, and this increase was significant. It is hypothesized that gout visits are on the rise due to increases in obesity, hypertension, and purine-rich diets [1, 2]. Obesity increases the production of serum urate (sUA) levels and also decreases urate excretion while weight reduction has been associated with uric acid level declination [16]. Along with risk factors, there are many disease associations with gout including metabolic syndrome, hypertension, and cardiovascular disease [16]. Metabolic syndrome has been strongly associated with gout; 60% of US population with gout also has metabolic syndrome, a prevalence three times higher in those with gout [17]. Metabolic syndrome has likely increased over the study years, helping explain the rise in gout diagnoses [18].

The number of gout visits in this study was not as high as noted in a similar prior study, [8] which could be attributed to several different factors. Although not specified in their methods, the Krishnan and Chen study appears to have used aggregated estimates for the years and databases studied, rather than the average annual estimates used in this study. Additionally, Krishnan and Chen only utilized NAMCS and NHAMCS-OPD, whereas this study also utilized NHAMCS-ED. Despite approximately 95 million visits attributed to the NHAMCS-ED, visits for gout in the ED were less likely. This likely increased the total number of overall visits without adding a commensurate number of gout-specific visits to the numerator. Further, while only 31% of the study population was aged 65 or older, 61% was female and the prevalence of gout is known to both increase with age and be more prevalent in males [3].

Another prior study with higher gout estimates by Zhu, et al. was based on participant reported data from the National Health and Nutrition Examination Survey (NHANES), which lends itself to estimating true prevalence [1]. This study is based on provider reported ambulatory, outpatient and emergency visits, therefore limiting the ability to estimate the true prevalence of gout. This database distinction helps explain this difference in gout estimates. It is worth noting that NHANES as well as the data sources used for this study are all population-based surveys.

The study results are consistent with several international epidemiology studies which examined the prevalence of gout [19–23]. The prevalence of gout has increased significantly in the United Kingdom (UK) over the years of 1997 through 2012 according to a study which utilized the Clinical Practice Datalink [19]. Another study which looked at gout prevalence in the UK and Germany from 2000 to 2005 with the IMS Disease Analyzer found a prevalence of 1.4% [20]. A study of the Canadian province of British Columbia from 2000 to 2012 using PopulationDataBC found a prevalence of 3.8% in 2012, and there was a noted increase over the study period [21]. A Swedish study examined gout trends from 2002 to 2012 and found a prevalence of 1.8% in 2012 as well as an increase over the study period. [22] A study in Taiwan utilizing the National Health Insurance Research Database found a higher prevalence rate of 6.24% over the study period of 2005 to 2010 [23]. With the exception of the Taiwan study [23], all studies were consistent with this study’s findings with regards to gout prevalence and increasing prevalence over the years.

This study demonstrated an association between age, sex, race group and gout visits, with an increased proportion among older age groups (≥45 years of age), males, African American and ‘Other’ race groups. These finding are consistent with previous studies [1, 3, 8, 10] showing that the risk of developing gout is age-related, [1, 8] and that estrogen is protective in premenopausal women due to its uricosuric effect [10]. ‘Other’ race within the databases consists of Asian, Native Hawaiian or other Pacific Islander, American Indian or Alaska Native, or more than one race reported. A higher prevalence of gout is well known in Asians and Pacific Islanders, as well as African Americans with genetics playing a role due to hyperuricemia-associated DNA sequence variations [24, 25]. However, diet and the presence of co-morbidities cannot be ruled out.

Hispanic/Latino individuals were found to be less likely to have a visit with gout than Non-Hispanic/Latinos. One possible explanation is related to diet. A previous study showed that Non-Hispanic/Latinos consume more red meat and seafood when compared to Hispanic/Latinos. [26] Diets rich in red meat and seafood are widely known to be associated with the production of uric acid [2]. Given that Hispanic/Latino diets are typically more heavily based on grains and beans along with fresh fruits and vegetables, Hispanic/ Latinos may produce less uric acid resulting in a lower incidence of gout [27].

Patient visits with Medicaid and ‘Other’ insurance were less likely to have a diagnosis of gout. Despite the lack of statistically significant interaction effects in the multivariable model, this could be attributed to the role of age with the risk of developing gout [1, 8]. ‘Other’ insurance consisted of worker’s compensation, self-pay, no charge/charity, and other, while Medicaid also included the Children’s Health Insurance Program. Patients with ‘Other’ insurance or Medicaid are less likely to be older, the age group at the highest risk for gout.

Individuals were significantly less likely to have a visit with a diagnosis of gout in a hospital emergency setting than they were in a physician’s office. Patients are more likely to visit a provider’s office for routine check-ups and for chronic conditions like gout. While individuals may visit a hospital for an initial or particularly severe attack of gout, they are presumably more likely to visit their provider when simply attempting to help keep their gout under control. Furthermore, patients with gout are much more likely to visit their provider if they are being prescribed gout prophylaxis medication.

A study by Garg, et al. looked at gout-related health care utilization in US emergency departments utilizing the National Emergency Department Sample (NEDS) from 2006 to 2008 [28]. The Garg study found approximately 0.7% of ED visits to be gout-related, slightly higher than 0.4% found in this study [28]. A similar study by Jinno, et al. also utilized NEDS and examined gout ED visits from 2006 to 2012 [29]. This study found 0.19% of visits with a primary diagnosis of gout [29]. Although not exactly comparable to this study, which includes non-ED databases in addition to the NHAMCS-ED, similar findings with both of these studies include gout-related ED visits being more likely with men, and increasing age; and less likely with different insurance types [28, 29].

Diuretic use was four times more likely to be associated with a gout visit. Previous research has shown that individuals who have high blood pressure and are also taking a diuretic have an increased risk for acquiring gout [6]. The diuretics’ mechanism of action is thought to contribute to gout, increasing uric acid reabsorption [30].

The graph of gout medication class by year showed consistency in use among the drug classes over the years. Antigout and antihyperuricemic medication classes remained the two most commonly prescribed treatments, while NSAIDs and steroids were used less. It is worth noting that due to drug class coding within the databases some medications could have been coded in both the antigout and antihyperuricemic class (i.e., allopurinol and febuxostat) since drugs may be coded in as many as four different medication classes. This might explain why the antigout percentage is greater than the antihyperuricemics. However, the findings in this study are consistent with the prior NAMCS and NHAMCS-OPD study which looked at gout treatment trends up through 2009 [8]. These treatment trends can also be explained by typical prescribing patterns for a gouty attack versus prophylaxis treatment to prevent gout flare. NSAIDs and steroids are typically only used for gouty attacks and patients are treated prophylactically after an initial gout attack to prevent future attacks [2, 31]. In addition, the risk of side effects with NSAIDs such as gastrointestinal bleeds, renal failure, and hypertension likely impacted their use in treatment, especially in the case when chronic treatment is warranted [32, 33].

As evident from the figure showing the percentage of visits by year for individual gout drugs, allopurinol continues to be the most prescribed treatment with colchicine second, a finding also consistent with Krishnan and Chen [8]. Allopurinol dominated the market as the only medication to reduce uric acid synthesis until the introduction of febuxostat in 2009 [2, 4, 11]. As expected, the percentage of visits with febuxostat increased following its approval. Despite this, allopurinol and colchicine use changed little from 2009 through 2011, evidence that febuxostat introduction had minimal impact on the treatment trends for the study years. Probenecid use has declined over the years which can be explained by its potential for drug-drug interactions as well as less favorable side effect profile, including risk of urolithiasis [2, 11, 34].

The previously mentioned international studies showed similar treatment trends. Allopurinol was prescribed for most patients in UK and Germany at 89 and 93% respectively; while colchicine use was only around 15–16% for both [20]. Probenecid use was minimal (< 1%), but NSAIDs were utilized 80–90% for prophylaxis. [20]. Allopurinol was also most commonly prescribed in British Columbia, Canada, with less than 1% use of febuxostat and probenecid [21]. Colchicine and steroid use increased in British Columbia over the study period, while NSAID use declined by 31% [21]. A study in Australia in 2005 found allopurinol to comprise 98.4% of all urate lowering therapy with probenecid at < 1% [35]. There was a common theme from these studies of the overall underutilization of urate-lowering treatment for gout [19–23, 35].

The study is not without limitations. The observational, cross-sectional nature of the study design limited the authors to statements of association between visits with gout diagnosis and the factors of interest. No claims of causality can be made. Furthermore, the cross-sectional nature of the data sources used did not allow for repeated measurements on patients over time. Several variables of interest, including alcoholism, Parkinson’s disease, depression, hypertension, weight status, tobacco use, and losartan use had to be excluded from all analyses due to missing data and/or reliability issues. This is particularly unfortunate for variables such as hypertension and weight status, both known to be significantly associated with gout. All of the databases utilized are limited to three diagnoses. The NAMCS and NHAMCS-OPD include a data field to collect other specific disease states (includes hypertension, diabetes, and depression), but NHAMCS-ED does not and only collects the diabetes variable of interest in their other specific disease field. This likely contributed to the missing data for such highly prevalent conditions like hypertension and diabetes. The NAMCS and NHAMCS databases do not include federal offices or hospitals, including Veterans Affairs facilities where gout can be prevalent. In addition, the databases do not provide a true prevalence of gout, but rather a surrogate via visits for gout based on diagnostic codes from the three recorded diagnoses and gout medications prescribed at the visits. It is not uncommon for epidemiological studies to rely on diagnostic codes for estimating prevalence. Those studies which have relied on such codes have shown good accuracy. In addition, although gout medications were also used to identify gout visits, there is a chance that medications like colchicine and probenecid were used for conditions other than gout. However, such alternative uses are rare. Study strengths include the use of nationally representative, population-based surveys which allow for generalizing findings to the portion of the US population that is commensurate with the study population. Further, the databases are provider reported data which allows for more reliability of results as compared to patient reported data. This is the first study known to the authors to investigate febuxostat in the treatment of gout since its approval in 2009 [4].

## Conclusion

This study found that the proportion of visits with a diagnosis of gout continues to increase, although the overall percentage of gout remains low in the US population. Individuals who are male, aged 45–64 or 65 and older, non-Hispanic/Latino, African American or of ‘Other’ race, use private insurance, present to a physician’s office, or use a diuretic are more likely to have a visit with a diagnosis of gout. Treatment trends over the study years by medication class and individual gout medications have remained consistent, with the introduction of febuxostat having little impact for the study years through 2011.

### Poster presentations

2016 Wiggins Academic Symposium, Campbell University, Buies Creek, NC.

2017 Interprofessional Health Sciences Research Symposium, Campbell University, Buies Creek, NC.

## Abbreviations

CIs: Confidence intervals
MSA: Metropolitan statistical area
NAMCS: National Ambulatory Medical Care Survey
NCHS: National Center for Health Statistics
NEDS: National Emergency Department Sample
NHAMCS-OPD/NHAMCS-ED: National Hospital Ambulatory Medical Care Survey Outpatient Department & Emergency Department
NHANES: National Health and Nutrition Examination Survey
ORs: Odds ratios
sUA: Serum urate

## Footnotes

1 The NAMCS and NHAMCS surveys define the four regions that comprise this variable as follows: Northeast: Connecticut, Maine, Massachusetts, New Hampshire, New Jersey, New York, Pennsylvania, Rhode Island, Vermont; Midwest: Illinois, Indiana, Iowa, Kansas, Michigan, Minnesota, Missouri, Nebraska, North Dakota, Ohio, South Dakota, Wisconsin; South: Alabama, Arkansas, Delaware, District of Columbia, Florida, Georgia, Kentucky, Louisiana, Maryland, Mississippi, North Carolina, Oklahoma, South Carolina, Tennessee, Texas, Virginia, West Virginia; West: Arizona, California, Colorado, Idaho, Montana, Nevada, New Mexico, Oregon, Utah, Washington, Wyoming, Alaska, Hawaii
